# Physiological and Pathological Functions of TMEM30A: An Essential Subunit of P4-ATPase Phospholipid Flippases

**DOI:** 10.1155/2023/4625567

**Published:** 2023-05-09

**Authors:** Jingyi Li, Yue Zhao, Na Wang

**Affiliations:** ^1^Key Laboratory of Medical Electrophysiology, Ministry of Education & Medical Electrophysiological Key Laboratory of Sichuan Province, Institute of Cardiovascular Research, Southwest Medical University, Luzhou, China; ^2^Clinical Medical Laboratory, Wenjiang Hospital of Sichuan Provincial People's Hospital, Chengdu, China

## Abstract

Phospholipids are asymmetrically distributed across mammalian plasma membrane. The function of P4-ATPases is to maintain the abundance of phosphatidylserine (PS) and phosphatidylethanolamine (PE) in the inner leaflet as lipid flippases. Transmembrane protein 30A (TMEM30A, also named CDC50A), as an essential *β* subunit of most P4-ATPases, facilitates their transport and functions. With TMEM30A knockout mice or cell lines, it is found that the loss of TMEM30A has huge influences on the survival of mice and cells because of PS exposure-triggered apoptosis signaling. TMEM30A is a promising target for drug discovery due to its significant roles in various systems and diseases. In this review, we summarize the functions of TMEM30A in different systems, present current understanding of the protein structures and mechanisms of TMEM30A-P4-ATPase complexes, and discuss how these fundamental aspects of TMEM30A may be applied to disease treatment.

## 1. Introduction

The cell membrane of eukaryotes is divided into two layers: the inner layer is rich in phosphatidylserine (PS) and phosphatidylethanolamine (PE), while phosphatidylcholine (PC) and sphingomyelin (SM) are more abundant in the outer layer [1]. The uneven distribution of these four components creates physical surface tension to bend the membrane, which is closely related to many life activities in eukaryotes. At present, three types of enzymes have been found to affect the distribution of phospholipids in the membrane: flippase, floppase, and scramblase [2, 3].

P4-ATPase [4] is a type of flippase, which locates in endoplasmic reticulum, Golgi apparatus, and plasma membrane and generates and maintains phospholipid compositional asymmetry in cell membranes by flipping lipids from the extracellular side of the plasma membrane or from the luminal side of internal organelles to the cytosolic side. Importantly, mammalian P4-ATPases exhibit strict substrate specificities, such as Dnf1, Dnf2, ATP8A1, ATP8A2, ATP11A, ATP11B, and ATP11C mainly flip PS [5, 6] and ATP8B1, ATP8B2, ATP10A, and ATP10B preferentially flip PC [7–10]. Accumulating *in vitro* evidences suggests that an *α* subunit of P4-ATPase forms heterodimeric complex with an accessory *β* subunit from the TMEM30 family of proteins, which consist of three members in most vertebrates (TMEM30A/B/C, also known as CDC50A/B/C) and two members in human (TMEM30A/B) [11, 12]. Under abnormal conditions, PS will be exposed and recognized by macrophages and induce apoptotic cells to be engulfed and platelet coagulation. PS exposure is also involved in many other biological processes, including myoblast and osteoclast fusion, and the immune response [13]. There are as many as 14 types of P4-ATPases (ATP8A1, ATP8A2, ATP8B1-B4, ATP9A, ATP9B, ATP10B-10D, and ATP11A-C) in mammalian cells, which make it difficult to study their physiological functions due to coexpression of multiple P4-ATPases in the same type of tissue/cells and their functional redundancy.

In contrast, the auxiliary subunit TMEM30 family has only three members, with TMEM30A as the predominant one. TMEM30A is most widely distributed in all tissues and various types of cells and highly expressed in brain, cerebellum, liver, kidney, spine, testicle, and other organs (https://www.proteinatlas.org/ENSG00000112697-TMEM30A/tissue). Recently, its high expression has also been found in the lung and identified as ACE2-independent SARS-CoV-2 receptor [14]. It is reported that TMEM30A interacts with 11 of the 14 mammalian P4-ATPases [15], attracting a great deal of research attention. The interaction between them is crucial for the transporting of P4-ATPase from the endoplasmic reticulum and the stability of the two subunits after the correct folding of P4-ATPase [16, 17]. However, the subcellular localization of the complex is determined by P4-ATPase rather than TMEM30A [18]. Some researchers indicated that TMEM30A may transport short-chain choline phospholipids into mammalian cells [19] and regulate the trafficking of amyloid-*β* precursor protein (APP) in endosomes [20, 21]. It is also worth noting that TMEM30A is suggested as the potential receptor for SARS-CoV-2 cell entry, but the mechanism needs further study [14, 22]. In recent years, the cryoelectron microscopy (cryo-EM) structures of the P4-ATPase–TMEM30 complexes were determined and indicated the direct interactions between them [9, 23]. In this review, we summarize current knowledge on the structure of the P4-ATPase–TMEM30A complexes and the important roles of TMEM30A in different systems.

## 2. Structures of the P4-ATPase–TMEM30A Complexes

**Figure 1:**
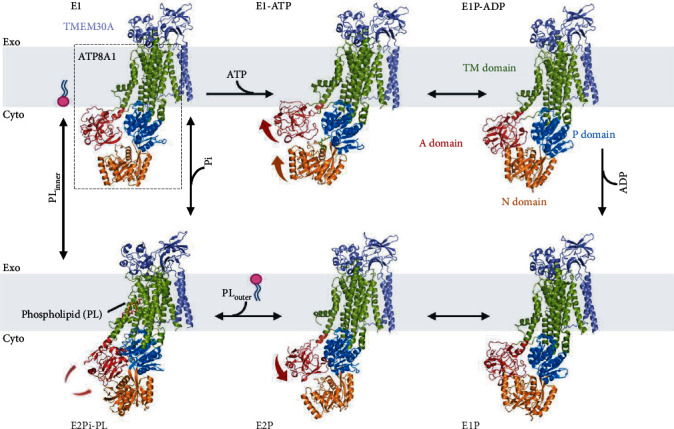
Phospholipid transport cycle of ATP8A1-TMEM30A flippase. Cell membranes are shown in gray, TMEM30A is shown in slate blue, ATP8A1 is boxed, and its four domains are shown in different colors (TM: transmembrane; A: actuator; P: phosphorylation; N: nucleotide-binding). The six intermediate states of ATP8A1-TMEM30A are arranged clockwise: E1, E1-ATP, E1P-ADP, E1P, E2P, and E2Pi-PL. Binding and hydrolysis of ATP trigger the transition of ATP8A1 from state E1 to E1-ATP and then to E1P-ADP. The subsequent phosphoryl transfer reaction induces the N and P domains proximally arrange after ATP binding at the N-domain, with a light outward shift of the A-domain. TMEM30A and the TM domain of ATP8A1 adopt almost the same conformation in all of these states. In E2P state, the N-domain is separated from the P domain and no longer accesses the phosphorylation site, while the A-domain is tightly fixed to the phosphorylation site, resulting in ADP-insensitivity. In the E2Pi-PL state, the P domain is phosphorylated, and phospholipid is released. Finally, ATP8A1 returns to the E1 state. Cyto: cytoplasmic side; Exo: exoplasmic side. PDBID: E1: 6K7H; E1-ATP: 6K7I; E1P-ADP: 6K7K; E1P: 6K7N; E2P: 6K7I; E2Pi-PL: 6K7M.

Early scientists discovered that TMEM30 proteins form heteromeric complexes with P4-ATPases [24, 25]. In 2019, Hiraizumi et al. [26] reported the first cryo-EM analysis of the human ATP8A1-TMEM30A heterocomplex in the six distinct intermediate states (Figure 1) at resolutions of 2.6 to 3.3 angstroms. The purified complex showed PS-dependent ATPase activity. The cryo-EM map reveals that ATP8A1 is composed of three large cytoplasmic domains (A: actuator; N: nucleotide-binding; P: phosphorylation) and ten membrane-spanning helices (M1-10), and TMEM30A has two transmembrane helices (TM1 and TM2) at the N- and C-termini, an ectodomain consisting of an antiparallel *β*-sandwich (*β*1-8), and extensions of about 60 and 70 amino acids with less secondary structure in the *β*3-4 and *β*5-6 loops, respectively. TMEM30A and ATP8A1 interact extensively through the extracellular transmembrane and intracellular regions, and the PS binding site is suggested to be located between the M1-2 and M3-4 segments. TMEM30A and the TM domain of ATP8A1 adopted very stable conformation throughout the transport cycle (Figure 1).

The crystal structure of human ATP11C-TMEM30A complex in the outward-open E2P conformation at 3.9 Å resolution was determined by Nakanishi et al. [27] in 2020. They found that the overall structure of the ATP11C-TMEM30A complex was very close to the corresponding E2P structures of previously reported human ATP8A1-TMEM30A complexes. The TM3-4 loop of ATP11C and the exoplasmic domain of TMEM30A formed a cavity, which captured PS at the exoplasmic side of the conduit. In 2022, a cryo-EM study on the ATP11C-TMEM30A complex obtained 3.4 Å and 3.9 Å structures of ATP11C transport intermediates and revealed a significant membrane protrusion in the vicinity of the end of TM2, which might be the phospholipid release site to the inner leaflet [28].

**Figure 2:**
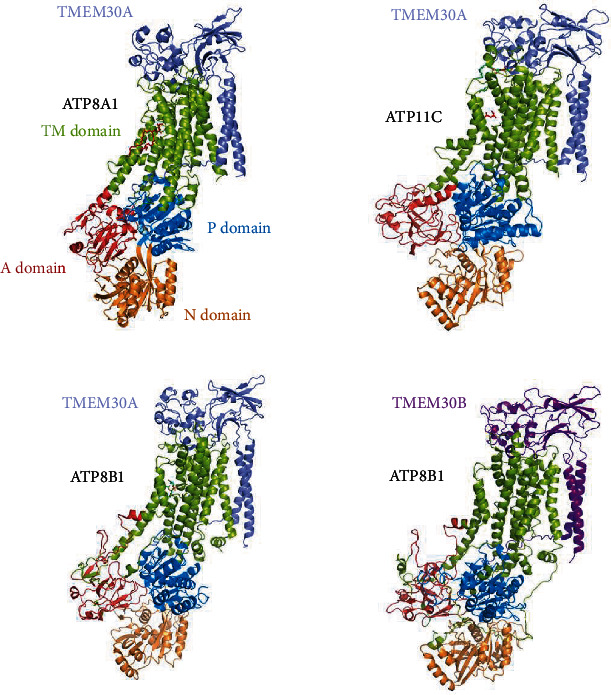
Cryo-EM structures of P4-ATPases-TMEM30A/B complexes. ATP8A1, ATP8B1, and ATP11C share a conserved structural arrangement typical of P4-ATPases. Each has a transmembrane (TM) domain and 3 cytoplasmic domains including A (actuator), N (nucleotide-binding), and P (phosphorylation) domains. TMEM30A and TMEM30B bind P4-ATPases in largely the same conformation, without notable structural differences. PDB ID: ATP8A1-TMEM30A: 6K7M; ATP11C-TMEM30A: 6LKN; ATP8B1-TMEM30A: 7VGJ; ATP8B1-TMEM30B: 7VGH.

The cryo-EM structures of human ATP8B1-TMEM30A/B were also disclosed in 2022 [23], in which the 3.4 Å structure of ATP8B1 was similar to that of ATP8A1. TMEM30A/B interacts with the extracellular domain of ATP8B1 through the two TMs and an unstructured loop preceding the N terminus of the TMs. At 4.0 Å resolution, PS bound at the cavity formed by TM2, TM4, and TM6 of ATP8B1 significantly activated the ATPase. TMEM30A and TMEM30B show the quite similar structure in both complexes (Figure 2). Another article in 2022 reported the 3.1 Å structure of human ATP8B1-TMEM30A [9]. The structure of TMEM30A was nearly identical to that previously observed (Figure 2). They further discovered the autoinhibition mechanism of ATP8B1 by its N- and C-terminal tails and demonstrated that the autoinhibition mechanism can be interfered with by exogenous peptides.

## 3. Nervous System

Some studies have implied the high expression in nervous system and involvement of ATP8A2 in brain functions and neurological disorders of patients [29, 30]. ATP8A2 is a member of the P4-ATPase family and works as a phospholipid transporter associated with its *β* subunit TMEM30A. Xu et al. [31] revealed for the first time that *Tmem30a* mRNA was highly expressed in cultured hippocampal neurons and might participate to regulate the axon length by ATP8A2. They also confirmed this on cultured PC12 cells *in vitro*. These results indicated the critical role of TMEM30A in enhancing neurite outgrowth.

In 2017, the retina-specific *Tmem30a* knockout mice were first generated to study its function *in vivo*. The data from 64 pups tested on P1 demonstrated that *Tmem30a* was essential for survival, and the protein was weakly expressed on photoreceptor cells. Nevertheless, the authors found that loss of *Tmem30a* in retinal neuronal progenitor cells resulted in the massive loss of neural cells and the degeneration of retina layers at P12. And the lack of *Tmem30a* in cones led to incorrect localization of cones and loss of photopic electroretinogram (ERG) response and cones. Deletion of *Tmem30a* in mouse embryonic fibroblasts resulted in decreased PS flippase activity and increased cell surface PS. Previous studies about *Atp8a2*-knockout mouse indicated that ATP8A2 was also located in photoreceptors, and its absence would lead to shortening of the outer segment of the photoreceptors and reduced photoresponse and photoreceptor viability in mice [32], while *Tmem30a* deficiency would not only lead to incorrect positioning of PS flippase ATP8A2 but also reduce the scotopic light response [33]. These differences indicated the distinctive physiological function of TMEM30A in the retina and its role in early embryonic development.

Purkinje cells (PC) are the only neurons in the cerebellar cortex capable of transmitting impulses, and *Tmem30a* Purkinje cell- (PC-) specific knockout (KO) mouse model was used to study the role of this gene in the cerebellum [34]. The experiment found that the *Tmem30a* KO mice exhibited early-onset ataxia and cerebellar atrophy, progressive PC death, and astrogliosis. The author further indicated that ER stress and subsequent cell apoptosis occurred in PCs lacking *Tmem30a*. It was noteworthy that the author did not detect that the expression of *Atp8a2* and *Tmem30a* might interact with other flippases in PCs. Further research is needed to explore the mechanism of TMEM30A in PCs.

Rod bipolar cells (RBCs) in the nervous system are second-order neurons in the retina that play an important role in transmitting visual information from rod cells and integrating into second-order neurons such as ganglion cells and amacrine cells [35]. Yang et al. [36] reported an RBC-specific *Tmem30a* knockout (cKO) mouse model with significantly compromised function of RBC and progressive RBC death. In the early degeneration process, RBC dendrites would also have significant compensatory synaptic remodeling, and accompanied by the migration of microglia to the outer plexiform layer to eliminate damaged synapses, this process would accelerate apoptosis and lead to the loss of RBCs. Collectively, *Tmem30a* was indispensable for the function and survival of RBCs.

Glial cells promote synaptic elimination through phagocytosis in the central nervous system during development and adult stages as well as in various neurological disorders. It is important to find out whether the recognition and regulation of “eat me” signals for subsequent synapses elimination are related to PS exposure by *Tmem30a*-assisted PS flippase. Park et al. [37] found that acute TMEM30A deletion in mature neurons led to preferential exposure of phosphatidylserine in neuronal soma and loss of specificity of inhibitory postsynapses, with no effect on other synapses, resulting in abnormal excitability and seizures. Phosphatidylserine was used for microglia-mediated inhibitory postsynaptic pruning in normal brains, suggesting that phosphatidylserine acts as a general “eat me” signal for inhibitory synaptic postsynaptic elimination. In the same year, Li et al. [38] further confirmed that TMEM30A was present at synapses in mice, and its expression was regulated by neuronal activity. TMEM30A knockdown caused phosphatidylserine exposure at synapses and induced aberrant synapse elimination by microglia *in vivo*. In addition, this study discussed that GPR56, as another receptor of phosphatidylserine other than MerTK, also participated in the regulation of TMEM30A knockdown-induced synaptic removal. In conclusion, these results both indicated that TMEM30A maintained synapses by regulating focal phosphatidylserine exposure at synapses, although its mechanism was still unclear.

In the pathogenesis of Alzheimer's disease (AD), it is increasingly believed that endosomal communication dysfunction precedes amyloid-*β* peptide (A*β*) and triggers AD [39, 40]. A laboratory in 2018 showed that TMEM30A was a candidate partner for *β*-carboxyl terminal fragments (*β*CTF) of amyloid-*β* precursor protein (APP), which physically interacted with *β*CTF in endosomes, impairing vesicle trafficking, resulting in abnormal enlargement of endosomes and impaired APP flow, and leading to the accumulation of APP-CTF, including *β*CTF [41]. They further reported in 2022 that the interaction between TMEM30A and *β*CTF was one of the important pathogenesis of Alzheimer's disease [20], which inhibited the physiological complex formation and activity of lipid flippase in SH-BACE1 cells, and the BACE1 inhibitor treatment recovered this interaction. In AD model mice, A7 and App^NL − G-F/NL − G-F^ knock-in model mice, they found TMEM30A/*β*CTF complex formation and subsequent lipid flippase dysfunction preceded A*β* deposition. These findings suggested that the interaction between TMEM30A and *β*CTF regulated vesicle transport through the asymmetric distribution of phospholipids, presented a therapeutic strategy for AD treatment.

## 4. Circulatory System

In mammals, hematopoietic stem cells (HSCs) are long lived and produce all other blood cells through the hematopoietic process to nourish the body and maintain its function. Li et al. [42] generated *Tmem30a*^−/−^ mice and found that *Tmem30a* deficiency in mouse bone marrow cells would markedly reduce the number of HSCs by downregulating the mammalian target of rapamycin (mTOR) signaling pathway, and the number of hematopoietic progenitor cells (HPC), white blood cells, B/T cells, and myeloid cells also decreased. The data revealed that *Tmem30a* was crucial to the survival of these cells, and even knockout mice could not survive for 50 days. Importantly, leukemia stem cells (LSCs) rapidly reduced before HSCs, which indicated that they were more sensitive to the *Tmem30a* deletion than HSCs and might suggest that *Tmem30a* was a potential target for the treatment of chronic myelocytic leukemia.

ATP11C is the only abundant P4-ATPase phospholipid flippase in human erythrocytes, while ATP11C and ATP8A1 are the main P4-ATPases in mouse erythrocytes. One study found that the loss of ATP11C phospholipid flippase activity combined with phospholipid hyperallergenase activity led to the exposure of phosphatidylserine on the surface of erythrocytes, reducing erythrocyte survival and leading to anemia [43]. It is well known that TMEM30A is an auxiliary subunit of P4-ATPase, so it is reasonable to speculate that the deletion of mouse *Tmem30a* may lead to abnormal function of ATP11C and ATP8A1, resulting in a decrease in the number of hematopoietic cells.

In the same year, it was found that knocking out *Tmem30a* in mouse hematopoietic cells caused severe anemia in their embryos on day 16.5, resulting in embryonic death, and a decrease in the number of erythroid colonies and erythroid cells in the liver of the fatal embryo, which may be due to impaired localization of erythropoietin-stimulating erythropoietin receptors in membrane raft microregions in *Tmem30a*-deficient cells [44]. In conclusion, *Tmem30a* regulates the EPOR signaling pathway through the formation of erythrocyte membrane rafts and plays a key role in erythropoiesis.

In the study of angiogenesis, retinal vascular production is the most representative, so Zhang et al. [45] studied the effect of TMEM30A in retinal endothelial cells. The study found that knocking down TMEM30A in human retinal endothelial cells *in vitro* would damage angiogenesis. Then, by crossing the *Tmem30a* conditional knockout allele with Pdgfb-CreER to generate an inducible vascular endothelial cell- (EC-) specific knockout line, it was found that the reduction of *Tmem30a* led to a decrease in vascular endothelial growth factor (VEGF) signal. As a result, endothelial cell proliferation was impaired, eventually delayed angiogenesis, and decreased tip endothelial cells and blunt-end aneurysm-like structures with fewer malformed filamentous pores in retinal blood vessels, but TMEM30A did not take effect on the maintenance of the vascular structure.

## 5. Motion System

Skeletal muscle is in the motor system, closely related to basic life processes such as respiration, metabolism, body temperature maintenance, and exercise, and its postinjury regeneration is mainly carried out by stem cells through asymmetric division and proliferation, which is inseparable from cell polarity. And *Tmem30a* has a great impact on cell polarity. It is reported that loss of myoblast *Tmem30a* in physiological states led to the loss of aminophospholipid flippase activity and impaired actin remodeling, RAC1 GTPase membrane targeting, and cell fusion [46]. However, deletion of the P4-ATPase ATP11A did not have a strong effect on cell fusion. This all suggested that myoblastic fusion was TMEM30A-dependent and might involve multiple TMEM30A-dependent P4-ATPases that help regulate actin remodeling. Under the pathological conditions of skeletal muscle injury, *Tmem30a* conditional knockout mice with Pax7^CreER^ tool represented the inhibition of the expression of the satellite cell proliferation regulator Pax7 and embryonic myosin heavy chain MYH3 in stem cells, affecting the proliferation process of stem cells. Previous studies have shown that the ATP11A-TMEM30A complex may affect myotube formation [47]. The researchers noticed this and investigated the role of TMEM30A in skeletal muscle regeneration by generating a satellite cell-specific TMEM30A conditional knockout mouse model [48]. They found that the deletion of *Tmem30a* also reduced the expression of muscle regulatory factor MRF, thereby affecting myoblast differentiation [48]. From the only two references, TMEM30A is an important factor in actin remodeling and skeletal muscle regeneration under both physiological and pathological conditions. But how to regulate actin remodeling still needs further investigation.

## 6. Digestive System

Insulin processing, maturation, and secretion are precisely regulated, and their dysregulation can lead to severe defects in glucose processing, leading to diabetes. ATP8B1 and TMEM30A were found to be highly concentrated in the insulin-secreting granule ISG in 2015, and gene silencing of a single P4-ATPase and TMEM30A inhibited glucose-stimulated insulin release in pure *β* cells and human islets [49]. However, the mechanism of action of TMEM30A in this was not yet clear. In a recent study, it was found that when *Tmem30a* was conditionally knocked out in mouse islet B cells, it would lead to an insufficient release of insulin, resulting in obesity, hyperglycemia, glucose intolerance, hyperinsulinemia, and insulin resistance in mice, and some mice would also spontaneously experience liver damage, which might be caused by disorders of glucose/lipid metabolism [50]. In addition, TMEM30A might play an important role in mediating vesicle transport between trans-Golgi apparatus (TGN) and plasma membrane (PM), and its lack could impair clathrin-mediated vesicle budding, hindering the maturation of insulin in immature secretory granules and the transport of glucose-sensitive Glut2 to PM [51]. The important role of TMEM30A in insulin maturation and glucose metabolism was elucidated.

Bile acids are synthesized in the liver and secreted into the intestine, and their homeostatic disorders can bring about pathological phenomena such as cholestasis. ATP8B1 mutations have been found to cause progressive familial intrahepatic cholestasis type 1 in humans, which was characterized by canalicular cholestasis [52]. As a homolog of ATP8B1, ATP11C-deficient mice were characterized by conjugated hyperbilirubinemia and unconjugated hypercholesterolemia, and functional studies have shown that hepatic uptake of unconjugated bile salts was severely impaired, and conjugated bile salt uptake was not affected. By using *Tmem30a* liver-specific knockout (LKO) mice, the researcher found that knockout of *Tmem30a* in the liver impaired the endoplasmic reticulum outlets of ATP8B1 and ATP11C, resulting in increased proteasome hydrolysis and hydrolysis of BS transport-related proteins. Also, the lack of *Tmem30a* reduced the activity of flippase, interfered with the phosphorylation of PKC*ζ*, and affected FXR*α* and other signaling pathways related to bile synthesis and transport, resulting in a decrease in the level of BS transporter (organic anion-transporting polypeptide OATP1A4, OATP1B2, sodium-taurocholate cotransporting polypeptide NTCP, etc.), and, eventually, hyperbilirubinemia and hypercholesterolemia [53]. Latest research also reported that the TMEM30A liver-specific knockout (LKO) mice showed severe liver damage, including macrophage infiltration and liver fibrosis caused by intrahepatic cholestasis. And DEN + TCPOBOP-induced hepatocellular carcinoma could happen in TMEM30A LKO mice [54]. So, TMEM30A plays an important role in intrahepatic bile homeostasis.

## 7. Urinary System

Glomerular epithelial cells (podocytes) are located at the outermost layer of the glomerular filtration barrier, whose damage and loss are currently thought to be the initiating cause of proteinuria and podocytopathy [55] (including focal segmental glomerulosclerosis (FSGS), minimal change disease (MCD), membranous nephropathy (MN), and diabetic nephropathy (DN)), as well as other types of kidney diseases (such as immunoglobin A nephropathy (IgAN) and lupus nephritis). As early as 2012, researchers found that TMEM30A localized to the apical region of proximal convoluted tubules of the cortex in the kidney [12], but it was not until 2021 that Liu et al. [56] had in-depth discussions on the relationship between TMEM30A and podocyte disease. They found that glomerular *Tmem30a* expression levels were significantly reduced in patients with microchange disease (MCD) and membranous nephropathy (MN). Then, they constructed podocyte-specific *Tmem30a* knockout mice using NPHS2-Cre mice to elucidate the role of *Tmem30a* in the kidney. *Tmem30a* KO mice showed significantly higher albuminuria levels compared with wild-type (WT) mice and podocyte injury and loss. Studies have revealed that in *Tmem30a* KO mice, small foot process (FP) formation was impaired, slit septum (SD) was deficient, and glomerular basement membrane (GBM) was increased which might be the cause of proteinuria, accompanied by increased expression of endoplasmic reticulum stress-related proteins such as CHOP and PDI, confirming endoplasmic reticulum stress, and these mice were experiencing focal segmental glomerular sexualization [56]. In summary, this research provides us with another unique perspective to understanding the mechanism of podocyte damage, and TMEM30A may be a potential target for treating podocyte diseases.

## 8. Immune System

As early as 2004, some scholars proposed that TMEM30A was interrelated to the progression of prostate cancer [57]. Later, Romanuik et al. [58, 59] detected the increase of TMEM30A transcript level in patients with primary prostate cancer, and its gene expression trend was consistent with the existence of proliferating cells in castration-recurrent prostate cancer (CRPC). Another article showed that overexpression of the TMEM30A initiated tumor cell migration by influencing downstream genes [60]. The role of TMEM30A in tumor cells is increasingly revealed.

Disseminated large B-cell lymphoma (DLBCL) is the most common subtype of lymphomas [61], but there is still a 40% chance of failure in current treatment; Ennishi et al. [62] found that biallelic TMEM30A loss-of-function mutation had a positive effect on the treatment of the disease in 347 patients with DLBCL. And this mutation was prone to aggressive B lymphoma (DLBCL, transformed follicular lymphoma, and primary central nervous system lymphoma). It was less likely to occur in Burkitt lymphoma, follicular lymphoma, and nonhematologic malignancies and rarely in other hematologic malignancies. In experiments on cell lines, an increase in BCR transfer rate and B-cell receptor signaling was found in TMEM30A-knocked-out related cells, which might be related to PS exposure by TMEM30A, as lipid rafts were rich in PS, and B-cell receptors were also localized in lipid rafts. At the same time, these cells were sensitive to chemotherapy drugs, and macrophages had increased phagocytosis, indicating that its deletion has a positive effect on tumor-related diseases, which is different from the function in other systems. Sworder et al. [63] had also found that TMEM30A affected the cellular microenvironment and was associated with cell resistance. van der Mark et al. [64] found that TMEM30A-depleted THP-1 macrophages had reduced tolerance to endotoxins.

## 9. Perspective

As an auxiliary subunit of P4-ATPase, TMEM30A is inseparable from the correct folding, stable expression, endoplasmic reticulum output, and enzyme activity of P4-ATPase [65]. The cryo-EM analyses confirm the structures of P4-ATPase complexes and the mechanisms about autoregulation, lipid specificity, and transport. In particular, the display of binding sites to their specific substrates in P4-ATPases provides direct evidence to reveal the function of the complex. TMEM30A and P4-ATPase always seemed to work together by forming heterocomplex and played an essential role in a wide range of cellular processes [66]. The deletion of *Tmem30a* would lead to cell death, even early embryo death, particularly resulting from PS abnormally exposed to the surface of lipid membrane. It makes the cells express “eat me” or apoptosis signals, and on the other hand, downstream signal pathway is activated by altering the localization of the receptor molecules in PS-rich lipid rafts, thus causing physiological events such as blood clotting [67].

The coronavirus pandemic since 2019 has become a hot topic in life science research. Studies have shown that SARS-CoV-2 can infect not only ACE2-rich cells but also cells lacking ACE2, and the infection is resistant to monoclonal antibodies against spike RBD *in vitro*, suggesting that some human cells have ACE2-independent alternative receptors that mediate SARS-CoV-2 entry [14]. TMEM30A, LDLRAD3, and CLEC4G have been identified as functional receptors for COVID [22], promoting the entry and spread of the virus, and may be the target of novel therapeutic agents [68].

To date, our understanding on the cellular function of TMEM30A is focused on its role as a chaperone of the P4-ATPases to facilitate their correct folding and trafficking. TMEM30A forms complexes with members of the ATP8A/ATP8B/ATP10/ATP11 family P4-ATPases and is essential for their functions at particular subcellular localizations [69]. As a single cell type usually expresses several different P4-ATPases that all assemble with TMEM30A, the phenotype of TMEM30A deficiency is largely a combinational effect of the dysfunction of multiple P4-ATPases, which is much more severe than deletion of an individual P4-ATPase [46]. So far, there is no evidence that TMEM30A has a function independent of P4-ATPases; however, it cannot be ruled out that TMEM30A may play a physiological role without associating with P4-ATPases, which should always be taken into consideration in future studies.
